# Adsorption Properties of Polyethersulfone-Modified Attapulgite Hybrid Microspheres for Bisphenol A and Sulfamethoxazole

**DOI:** 10.3390/ijerph17020473

**Published:** 2020-01-11

**Authors:** Jian Yu, Hao Shen, Bin Liu

**Affiliations:** Key Laboratory of Building Safety and Energy Efficiency, Ministry of Education, Department of Water Engineering and Science, College of Civil Engineering, Hunan University, Changsha 410082, China; jianyu@hnu.edu.cn (J.Y.); haoshen@hnu.edu.cn (H.S.)

**Keywords:** endocrine disruptor, bisphenol A, sulfamethoxazole, attapulgite, adsorption

## Abstract

In this paper, attapulgite purified by acid heat was employed, and millimeter polyethersulfone-modified attapulgite hybrid microspheres were prepared. The effects of mixed bisphenol A (BPA) and sulfamethoxazole (SMX) solution on the modified attapulgite doping ratio, initial solution pH, adsorbent dosage, contaminant concentration, and temperature were studied. The experimental results showed that BPA and SMX compete in the adsorption process, and the hybrid microspheres preferentially select the BPA molecules (anionic endocrine disruptors) compared to the SMX. The adsorption process in the mixed solution conforms to the quasi-secondary-order kinetic model. The adsorption of BPA and SMX by hybrid microspheres is more consistent with the extended Langmuir adsorption thermodynamic model, and the adsorption of BPA + SMX is more in line with the Langmuir adsorption thermodynamic model. At 25 °C, the maximum adsorption capacity of hybrid microspheres for BPA and SMX was 102.04 and 12.80 μmol·g^−1^, respectively, and the maximum adsorption of BPA + SMX was 112.36 μmol·g^−1^. After five regenerations, the removal effect of the hybrid microspheres on the endocrine disruptors remained above 95%.

## 1. Introduction

Endocrine-disrupting compounds (EDCs) are a class of hazardous substances that affect specific levels of endocrine (hormone) systems. Researchers, from the perspective of in vivo models and combined with clinical epidemiology, found that EDCs can cause diseases such as breast cancer and prostate cancer and affect human reproduction, thyroid, cardiovascular effects, and neuroendocrine systems [1]. In addition, EDCs can cause some metabolic diseases including obesity, diabetes, etc. EDCs can affect various functions of the body through different destruction pathways, for example, activation or inactivation of endocrine target receptors, disruption of hormone synthesis, activation of inactive hormones, etc. Studies showed that EDCs could affect the ability of hormone-metabolizing enzymes and affect the reactivity of nuclear receptors by altering hormone activity [2], change the normal course of estrogen and androgen action, inhibit or interfere with the synthesis of metabolic steroids [3], and influence estrogen and androgen receptors, promoting or inhibiting transcriptional or post-transcriptional synthesis mechanisms [4].

EDCs are usually organic pollutants or heavy metal substances. Most of the pesticides and stabilizers and plasticizers in plastics use endocrine disruptors, such as organochlorine pesticides, polychlorinated biphenyl (PCB), alkylphenol (AP), bisphenol A (BPA), alkylphenol ethoxylate (APE), and phthalate acid esters (PAE). EDCs come from a wide range of sources and can be discharged into natural water through certain channels, resulting in water pollution [5].

At present, EDCs can be found in major lakes and rivers in China [6]. Some drinking water sources and tap water were also detected as containing EDCs. The southern region is dominated by organic chemical raw materials such as polychlorinated biphenyls, BPA, and PAE. A variety of EDCs were detected in natural waters in the Yangtze River Delta, such as BPA in the Huangpu River (mass concentration 3.2–9.9 ng/L), BPA in Taihu Lake (mass concentration 262.39–1442.72 ng/L), polychlorinated biphenyls in Yangtze River and Taihu Lake (mass concentration 0–152 ng/L), sulfa antibiotics at the mouth of the Yangtze River (mass concentration 0.6–1613 ng/L), and phthalic acid in the effluent of water supply plants using Taihu Lake and the Yangtze River as water sources (mass concentration 0–6000 ng/L), which exceed the drinking water standard [6].

The removal methods of EDCs in sewage wastewater mainly include adsorption separation methods, membrane separation methods, advanced oxidation methods, and biodegradation methods. As a cost-effective water treatment technology, adsorption separation technology occupies an irreplaceable position with its mature practical application experience [7]. The adsorbent usually adsorbs pollutants onto its surface with the interaction of ion exchange, van der Waals force, electrostatic adsorption, etc. The adsorption method is mainly used for the removal of organic pollutants [8] and heavy metal contaminants [9]. Compared with other water treatment technologies, the adsorption method has the advantages of high removal rate, simple design, and easy operation. Meanwhile, the regeneration efficiency and waste formation still need to be resolved when applying the adsorption process. Lu et al. used fresh canola to modify nitrogen-doped carbon and found that it can effectively increase the absorption of BPA [10]. Solak et al. found that highly crosslinked polymer adsorbents have better removal rates of EDCs in municipal wastewater than activated carbon [11]. Chen et al. used cetyltrimethylammonium bromide to prepare organic attapulgite, which can effectively improve the adsorption of Congo red dye [12]. Lu et al. found that the attapulgite iron-oxide magnetic composite prepared by coprecipitation has good adsorption efficiency for heavy metal cerium(III) in water, and the composite material can be recovered by magnetic separation method [13]. Xi et al. used octadecyltrimethylammonium bromide and bisdimethylammonium bromide ion to modify the attapulgite and studied their maximum adsorption capacity of the anionic herbicide 2,4-dichlorophenoxyacetic acid [14].

Polyethersulfone (PES) is an amorphous polymer, which has the advantages of good resistance to steam and hot water, good water absorption, good dimensional stability, and excellent chemical resistance to acids and alkalis condition [15]. Liquid–liquid separation can be used to form PES millimeter-sized spherical particles with rich voids when dissolved it into *N*,*N*-dimethylacetamide solvent (DMAC). The PES material and the modified attapulgite were selected to make hybrid microspheres to adsorb the mixed solution of two endocrine disruptors (BPA and SMX). BPA and SMX are frequently detected in wastewater treatment influents; BPA can induce obesity, diabetes, and coronary heart disease, whereas SMX can lead to adverse microbial activity performance [16]. The adsorption principle of hybrid microspheres on endocrine disruptors in anion and cation forms in aqueous solution is of great significance for the further exploration of such hybrid microspheres and their application in water treatment.

## 2. Materials and Methods

### 2.1. Experimental Materials and Instruments

The attapulgite ore was obtained from the Xuyi deposit in Jiangsu Province, and its chemical composition is Mg_5_Si_8_O_20_(OH)_2_(OH_2_)_4_·4H_2_O. The structure is a layered chain, and the channel cross-section is about 0.37 × 0.63 nm. The purity of PES (PES, Ultrason E6020P, CAS Number: 25,608-63-3 Germany BASF) was above 99%. Experimental agents such as BPA, *N*,*N*-dimethylacetamide (DMAC), and cetyltrimethylammonium bromide (CTMAB) were purchased from Shanghai Aladdin Biochemical Technology Co. Ltd. and were of analytical grade.

BPA and SMX solutions were scanned at a wavelength range of 200 nm to 450 nm using a U-3900 ultraviolet–visible light (UV–Vis) spectrophotometer. When the wavelength of the spectrophotometer is 283 nm, the absorbance and concentration of BPA have a good linear relationship, while the absorbance and concentration of SMX have a good linear relationship at 296 nm. Moreover, in the mixed solution of BPA and SMX, when a linear multivariate regression equation is used, the linear relationship between the absorbance and concentration of each component is good. The correlation coefficient *R*^2^ of the regression equation is >0.99.

The hybrid microspheres were characterized by scanning electron microscopy (Hitachi, Tokyo, Japan, S-4800) and a specific surface meter (Conta Autosorb IQ, Shanghai, China).

### 2.2. Preparation of PES-Modified Attapulgite Hybrid Microspheres

#### 2.2.1. Attapulgite Purification

The attapulgite ore was ground to 100 mesh, and the mixture was stirred in the DMAC solution for 24 h. The upper suspension was centrifuged after precipitation, and the separated material was dried and ground to 100 mesh to obtain purified attapulgite.

#### 2.2.2. Preparation of PES-modified Attapulgite Hybrid Microspheres

*Acid heat modification treatment*: the purified attapulgite was immersed in 2 mol·L^−1^ hydrochloric acid in a water bath for 70 min, and the precipitate was taken out by suction filtration, then washed repeatedly with distilled water until neutral. After roasting for 2 h in a muffle furnace (200 °C), it was then sieved with a 100-mesh sieve to obtain an acid heat-modified attapulgite (AH-ATT).

*Organic modification treatment*: firstly, 5 g of CTMAB and 10 g of AH-ATT were dispersed into an ethanol/water (1000 mL/50 mL) mixed solution and stirred for 24 h. Then, the modified attapulgite was repeatedly resuspended, filtrated, and washed until the supernatant of the resuspension did not contain bromide ions. The organic acid heat-modified attapulgite (OAH-ATT) was obtained after drying and sieving (100 mesh).

*Preparation of hybrid microspheres*: PES was dissolved in DMAC and used to set up a solution with a mass fraction of 12%. OAH-ATT was added to the solution; then, the mixed solution was dropped into distilled water at a rate of 60 to 100 drops·min^−1^ at room temperature to prepare hybrid microspheres. The prepared microspheres were washed with distilled water, then washed with ethanol, and dried for use.

### 2.3. Calculation Method of Endocrine Disruptor

When the wavelengths of the spectrophotometer were 283 and 296 nm, the absorbances of the two endocrine disruptors of BPA and SMX in the solution did not interfere with each other based on the configured concentration of the mixed solution. Therefore, multiple linear regression methods can be used for analysis. In statistics, multiple linear regression is a method of fitting experimental data using linear equations to simulate the relationship between two or among more independent variables. Multiple linear regression can be expressed as follows [17]:

(1) $$Y=b_{0}+b_{1}X_{1}+b_{2}X_{2}+\cdots +b_{n}X_{n},$$

where Y is the target value, and the data are the total absorbance of the mixed solution of the spectrophotometer at the selected wavelength; X_n_ (n = 1, …, n) is the value of each independent variable, which is the absorbance of the two components of BPA and SMX at the selected wavelength; b_n_ (n = 1, …, n) is the coefficient of the regression equation. In the experiment, the mixed solution was configured with ultrapure water, and there was no background interference to the total absorbance, while other coefficients were calculated using the experimentally prepared standard solution.

### 2.4. Experiment on the Adsorption Effect of Hybrid Microspheres on Double Mixed Solution

Firstly, 0.075 g of hybrid microspheres were weighed into a mixed endocrine disruptor solution of 20 mL, which contained 25 μmol·L^−1^ BPA and 25 μmol·L^−1^ SMX. It was shaken for five days in a shaking incubator at 25 °C. The effect of doping ratio was determined (OAH-ATT:PES ratios of 0:1, 0.5:1, 1:1, 1.5:1, and 2:1, sequentially named PA1, PA2, PA3, PA4, and PA5, respectively).

In order to study the adsorption kinetics, hybrid microspheres with a doping ratio of 1:2 were added to the mixed solution at 25 °C, 200 rpm, and pH 6. The total concentration of the mixed solution was 50, 100, 150, 200, or 250 μmol·L^−1^. In order to study the adsorption thermodynamics, hybrid microspheres with a doping ratio of 1:2 were added to the mixed solution at a rotation speed of 200 rpm and pH 6 (the total concentration of the mixed solution was 50–500 μmol·L^−1^). The adsorption performance of hybrid microspheres at 25 °C, 40 °C, and 55 °C was investigated.

Considering that the concentrated liquid produced by acid–base regeneration is difficult to handle, pure ethanol was used as the regeneration liquid. Hybridized microspheres with a doping ratio of 2:1 were added to the mixed solution (20 mL, pH 6, 50 μmol·L^−1^ endocrine disrupting solution) and shaken for five days in a shaking incubator at 25 °C, 200 rpm. The adsorbed and equilibrated hybrid microspheres were placed into a 20-mL ethanol solution and regenerated in a shaking incubator at 25 °C, 200 rpm for one day. After that, it was washed repeatedly with ultrapure water at least three times and dried. The adsorption experiment was repeated again.

For the regeneration test, ethanol solution was utilized to regenerate the adsorbent. The adsorption amount (C) was detected after every regeneration process, and the initial adsorption amount was denoted as C_0_. The regeneration rate was equal to C/C_0_.

All of the above experiments were repeated three times, and the data were averaged.

## 3. Results and Analysis

### 3.1. Apparent Characteristics of Hybrid Microspheres

Diameter, porosity, and total pore volume of hybrid microspheres [18] were calculated according to Equations (2)–(4).

Diameter:

(2) $$D={\left(\frac{6(W_{A}\left(1-C\%\right)/\rho_{P}+W_{A}C\%/\rho_{C}+\left(W_{B}-W_{A}\right)/\rho_{W})}{n\pi }\right)}^{\frac{1}{3}},$$

Porosity:

(3) $$P=\frac{\left(W_{B}-W_{A}\right)/\rho_{W}}{W_{A}\left(1-C\%\right)/\rho_{P}+W_{A}C\%/\rho_{C}+\left(W_{B}-W_{A}\right)/\rho_{W}}\times 100\%,$$

Total pore volume:

(4) $$V_{P}=\frac{n\pi D^{3}P}{6W_{A}},$$

where *W_A_* is the mass of hybrid microspheres after drying (g), *W_B_* is the mass of hybrid microspheres before drying (g), *ρ*_w_ is the density of water (*ρ*_w_ = 1.0 g·cm^−3^), *ρ*_p_ is the density of PES (*ρ*_p_ = 1.43 g·cm^−3^), *ρ*_c_ is the density of modified attapulgite (*ρ*_c_ = 1.67 g cm^−3^), *n* is the number of weighed hybrid microspheres selected (*n* = 25), and *C* is the proportion of modified attapulgite in the hybrid microspheres (%).

The calculation results of the diameter, surface area, porosity, and total pore volume of the hybrid microspheres are shown in Table 1. In Table 1, as the doping amount of the modified attapulgite increased, the porosity and total pore volume of the hybrid microspheres gradually decreased, but the specific surface area gradually increased.

**Figure 1:**
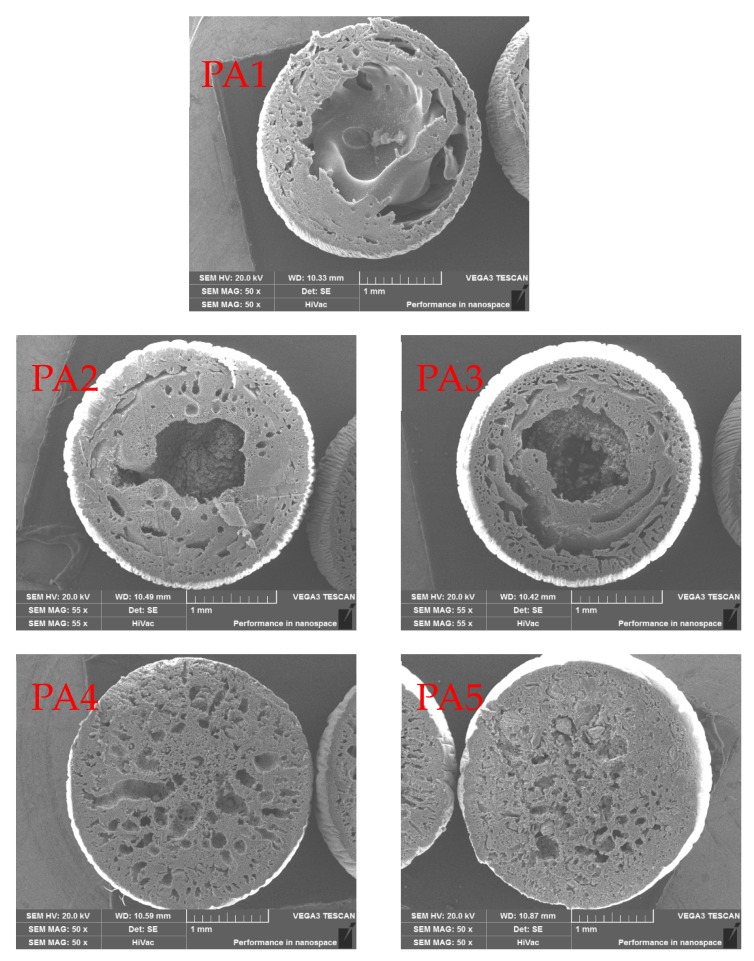
Scanning electron microscopy images of hybrid microsphere.

Figure 1 shows SEM images of hybrid microspheres. The characteristics of the hybrid microspheres in the figure were as follows: (1) pure PES microspheres formed an intermediate gap during the ball formation process, and the PES material wrapped the intermediate gap into a ball; (2) with the increase in doping amount of the modified attapulgite, the voids inside the hybrid microspheres were gradually reduced; (3) during the doping process of the modified attapulgite, the voids at the edge of the microspheres were preferentially filled, and the internal voids of the microspheres were gradually filled when increasing the doping amount; (4) by comparing PA4 and PA5, the macropores in the microspheres were filled when the doping amount continuously increased, and the modified attapulgite was filled in the small pores of the microspheres, thereby making the microspheres more compact.

### 3.2. Effect of Modified Attapulgite Doping Ratio

**Figure 2:**
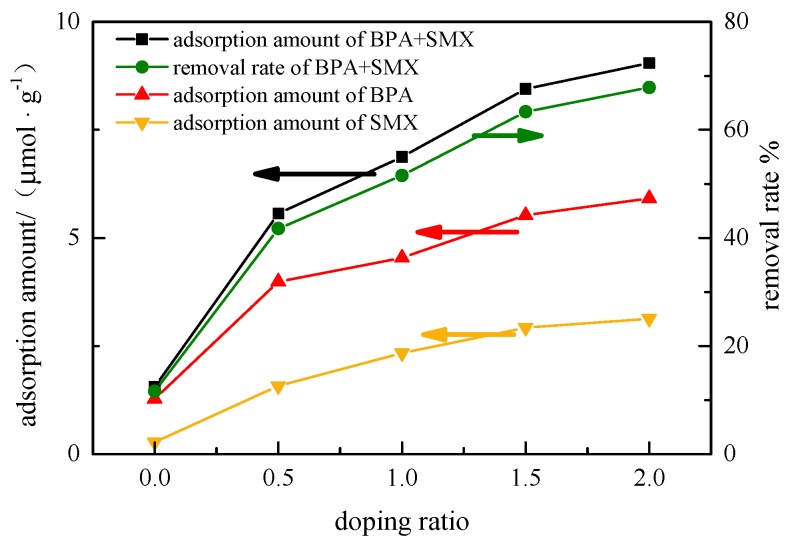
Effect of doping ratio of modified attapulgite on adsorption performance.

In Figure 2, when the doping ratio increased from 0:1 to 2:1, the adsorption amount of the hybridized microspheres to the BPA increased from 1.27 μmol·g^−1^ to 5.91 μmol·g^−1^, and the adsorption amount of SMX increased to 3.13 μmol·g^−1^ from 0.27 μmol·g^−1^. The removal rate of BPA + SMX increased from 11.62% to 67.85%. The pure PES microspheres had a poor adsorption effect on the endocrine disruptors BPA and SMX in the mixed solution, with only 1.27 μmol·g^−1^ and 0.27 μmol·g^−1^. The modified attapulgite played a major role in the adsorption process of endocrine disruptors, while the PES acted as a skeleton to support the modification of the attapulgite particles.

When the doping ratio of the hybrid microspheres increased from 0:1 to 1.5:1, the adsorption amount of the endocrine disruptors increased significantly by the hybrid microspheres, and the total adsorption amount increased from 1.55 μmol·g^−1^ to 8.45 μmol·g^−1^. However, when the doping ratio increased from 1.5:1 to 2:1, the adsorption capacity of the hybrid microspheres to the endocrine disruptors slightly increased. The total adsorption increased from 8.45 μmol·g^−1^ to 9.05 μmol·g^−1^. The adsorption active site increased in size due to an increase in specific surface area at a lower doping ratio, but further decreased in terms of porosity and total pore volume when the doping ratio of the hybrid microspheres was further increased. Thus, the increase in specific surface area did not effectively enlarge the adsorption active site. With the same doping ratio, the adsorption amount of BPA and SMX was lower when compared with the BPA in the single-component solution, but higher than with the SMX in the single-component solution. Overall, increasing the doping ratio could highly improve the adsorption amount of BPA and SMX.

### 3.3. Effect of the Initial pH of the Solution

**Figure 3:**
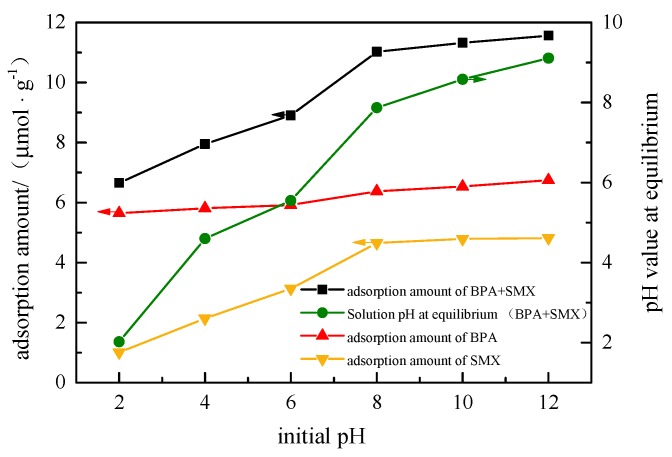
Effect of initial pH of solution on adsorption performance.

In Figure 3, as the pH of the solution increased from 2 to 12, the adsorption amount of BPA by the hybrid microspheres increased from 5.65 μmol·g^−1^ to 6.75 μmol·g^−1^, and the adsorption amount of hybrid microspheres to SMX increased from 1.00 μmol·g^−1^ to 4.81 μmol·g^−1^. This suggests that the electrostatic force had little effect on the adsorption process under alkaline conditions. The effect of CTMAB on the surface of the attapulgite had a greater influence on the adsorption capacity of the hybrid microspheres. Hybrid microspheres had a stronger adsorption effect on anionic endocrine disruptors than cationic ones. The adsorption effect of the hybrid microspheres on BPA and SMX could be effectively enhanced due to the ionization. This is probably because the pKa of BPA (9.6, 10.2) is much higher than that of SMX (1.8, 5.7) [19]. A previous study also reported that the adsorption amount of BPA was only slightly varied whether in acidic or basic environment [20].

### 3.4. Effect of Adsorbent Dosage

**Figure 4:**
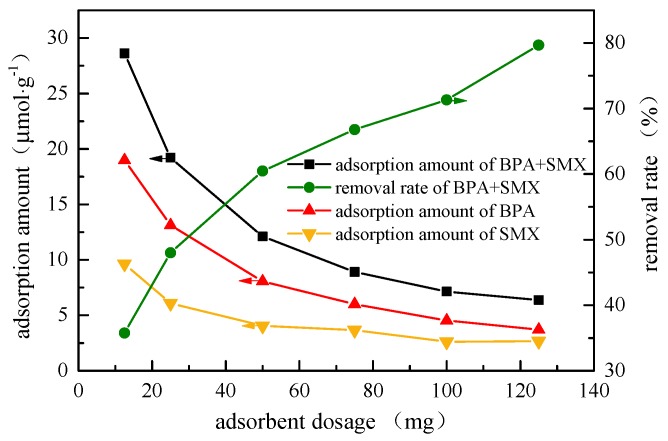
Effect of adsorbent dosage on adsorption performance.

In Figure 4, when the adsorbent dosage increased from 12.5 mg to 125 mg, the adsorption amount of the hybridized microspheres to the BPA decreased from 18.97 μmol·g^−1^ to 3.71 μmol·g^−1^, and the adsorption amount of SMX decreased from 9.64 μmol·g^−1^ to 2.66 μmol·g^−1^. The increase in adsorbent dosage could increase the effective active sites of the two endocrine disruptors, but the adsorption amount per gram decreased. Hybrid microspheres had a better affinity for hydrophobic endocrine disruptors, and the adsorption amount of BPA was higher than that of SMX. The lower adsorption amount of SMX was probably because the log K_ow_ of SMX (0.9) is much lower than that of BPA (2.2) [21].

### 3.5. Effect of Initial Endocrine Disruptor Concentration

**Figure 5:**
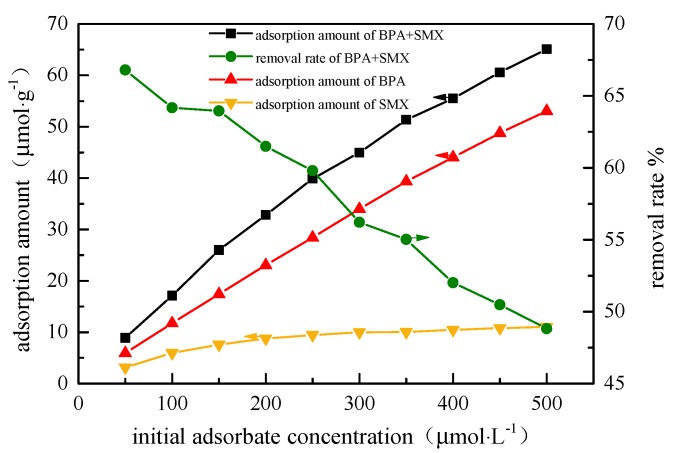
Effect of initial concentration of solution on adsorption performance.

In Figure 5, when the initial concentration increased from 50 μmol·L^−1^ to 500 μmol·L^−1^, the adsorption amount of the hybridized microspheres to the BPA increased from 3.13 μmol·g^−1^ to 11.04 μmol·g^−1^, and the adsorption amount of SMX increased from 5.105 μmol·g^−1^ to 53.05 μmol·g^−1^. Since the initial concentration of endocrine disruptors provides an important driving force to overcome the mass transfer resistance between the aqueous solution and the solid phase, the increase in endocrine disruptors concentration could increase the adsorption capacity of the adsorbent. However, since there is also a competition effect between the endocrine at the effective active sites, the removal effect of the hybrid microspheres deteriorated. The adsorption amount of the hybrid microspheres was lower than that in the unit system. This is due to the competitive relationship between BPA and SMX in the mixed system. By comparing the binary system and the unit system at the same concentration, the hybrid microspheres preferentially adsorbed BPA. The results are probably due to the higher negative charge of SMX than BPA, while some of the SMX adsorption sites were occupied by BPA molecules [22].

### 3.6. Adsorption Kinetics

**Figure 6:**
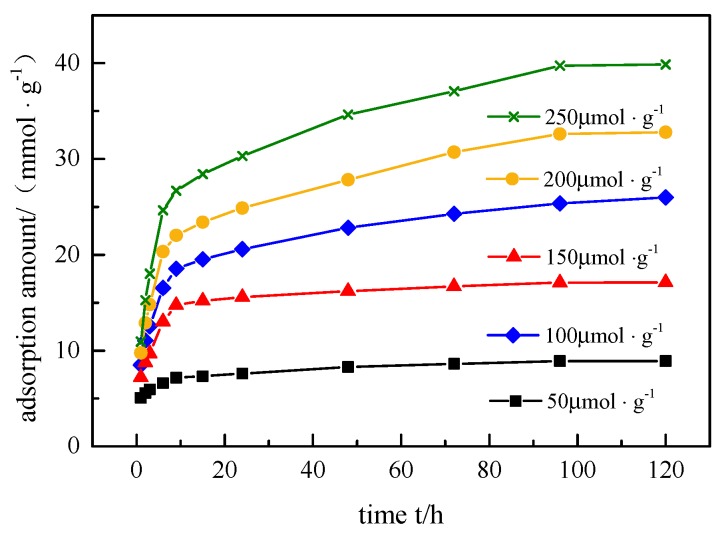
Effect of bisphenol A (BPA) + sulfamethoxazole (SMX) adsorption time on adsorption performance.

Figure 6 shows the adsorption kinetics of BPA and SMX. With the increase in initial concentration of the mixed solution, the adsorption amount of the hybrid microspheres to BPA and SMX also increased. The initial concentration of the solution provides an important driving force to overcome its mass transfer resistance between the aqueous solution and the solid phase. Therefore, a higher initial concentration of the mixed solution led to a stronger adsorption effect, that is, a larger adsorption amount of the hybrid microspheres to BPA and SMX.

The quasi-first-order kinetic model, quasi-second-order kinetic model, and intraparticle diffusion model were used to fit the process of adsorption of endocrine disruptors by hybrid microspheres. The results are shown in Table 2 and Table 3. In Table 2, the quasi-second-order kinetic model had a higher correlation coefficient (*R*^2^ > 0.996) relative to the quasi-first-order kinetic model (*R*^2^ < 0.962), and the theoretically calculated adsorption amount agreed well with the experimental adsorption amount, indicating that the adsorption process conformed to the quasi-secondary kinetic model. The q_e_ value increased with the initial concentration, while the rate constant (K_2_) decreased, due to the adsorption provided by a certain amount of adsorbent. The adsorption competition of endocrine disruptors on hybrid microspheres was less at lower concentrations, while the adsorption of endocrine disruptors at higher concentrations was more competitive.

The fitting results of the intraparticle diffusion model to the adsorption process showed that q_t_ and t^0.5^ exhibited multiple sets of linear relationships. The fitting data are shown in Table 3. The adsorption process could be divided into two stages. In phase I, the adsorption process was faster, and the removal rate of BPA + SMX accounted for about 60% of the total removal rate within 6 h. At this stage, macropore diffusion dominated. BPA and SMX were rapidly adsorbed onto the active sites at the outermost layer of the macropores. In phase II, the adsorption process proceeded slowly, and the hybrid microspheres reached the final adsorption equilibrium in about four days. BPA + SMX accounted for about 40% of the total removal rate. After the outer active site was saturated, the BPA and SMX molecules entered the active sites in the micropores, and intraparticle diffusion played a dominant role. The adsorption experiments were carried out at different stirring speeds, and it was found that the k_p_ value of phase I did not change much, which proved that phase I was not affected by the diffusion of the boundary layer of the particles. Therefore, during the process of endocrine disruptors in the hybrid microsphere, phase I is a macroporous diffusion process and phase II is an intraparticle diffusion process.

In Table 2 and Table 3, q_e,exp_ is the equilibrium adsorption amount obtained in the experiment, and q_e,cal_ is the calculated equilibrium adsorption amount.

### 3.7. Adsorption Thermodynamics

**Figure 7:**
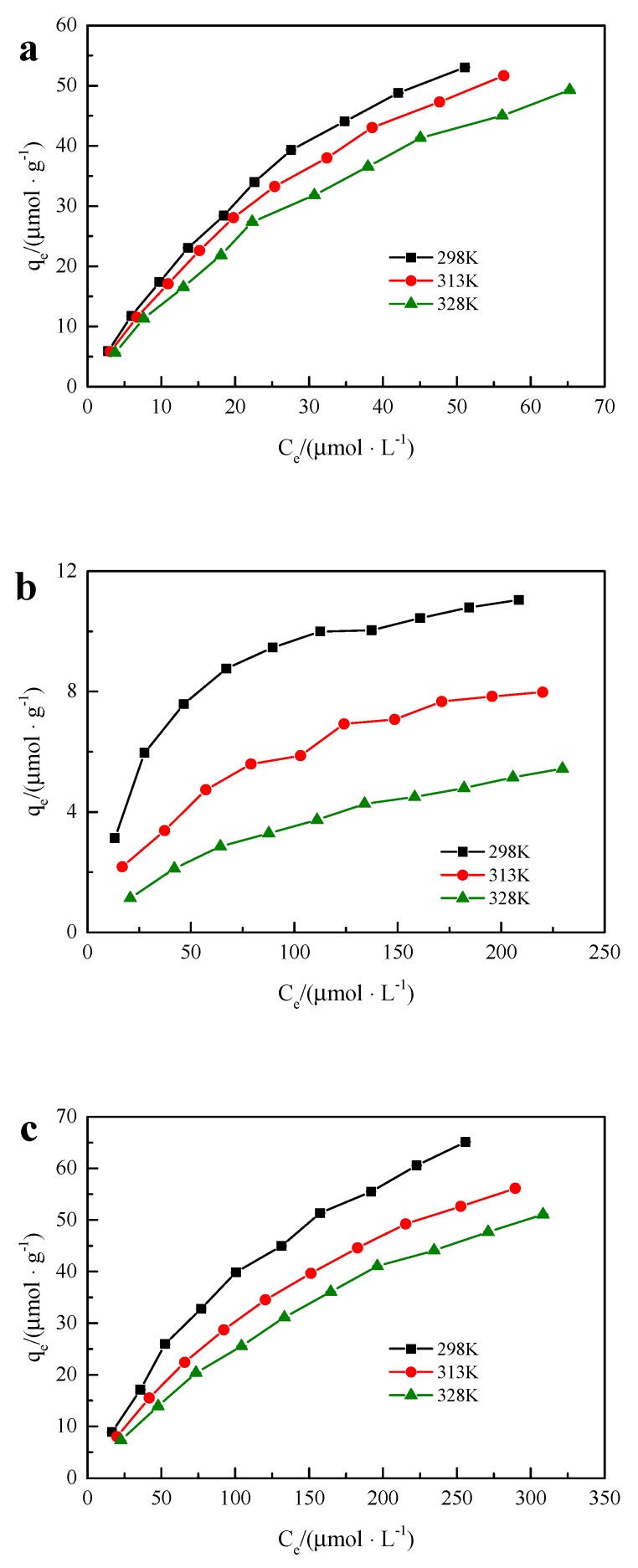
Adsorption isotherm in mixed solution: (**a**) BPA; (**b**) SMX; (**c**) BPA + SMX.

Different concentrations of the two-component solution were adsorbed by hybrid microspheres at temperatures of 298 K, 313 K, and 328 K. The adsorption isotherms of BPA, SMX, and BPA + SMX in the mixed solution of hybrid microspheres are shown in Figure 7. The adsorption amount of hybrid microspheres increased with the initial concentration of endocrine disruptors.

The Langmuir and Freundlich thermodynamic fittings were applied to the adsorption isotherms of BPA, SMX, and BPA + SMX. Multiple linear regression analysis methods were used to extend the Langmuir thermodynamic fitting of the BPA + SMX adsorption isotherms. All parameters after fitting are shown in Table 4. According to the fitting results, the adsorption process of BPA and by hybrid microspheres was more consistent with the extended Langmuir adsorption thermodynamic model, and the adsorption process of BPA + SMX was more in line with the Langmuir adsorption thermodynamic model. The fitting results show that the adsorption process was uniform monolayer surface adsorption.

The maximum adsorption capacities of hybrid microspheres in the mixed solution were 102.04 μmol·g^−1^, 97.09 μmol·g^−1^, and 90.91 μmol·g^−1^ under the conditions of 298 K, 313 K, and 328 K, respectively, while the maximum adsorption capacities of SMX were 12.80 μmol·g^−1^, 10.73 μmol·g^−1^, and 8.35 μmol·g^−1^. The adsorption amount of the hybrid microspheres to the SMX was lower than that of the SMX in the single-component solution, but the adsorption amount of the BPA in the mixed solution was close to that of the single-component solution. This indicated that the adsorption of SMX by the hybrid microspheres was greatly affected by the BPA in the mixed solution, while the adsorption of BPA in the mixed solution was less affected by the SMX in the mixed solution. This may be due to the different solubilities of BPA and SMX in water. The active adsorption sites of hybrid microspheres were limited, and BPA molecules with lower solubility were preferentially selected in the mixed solution of BPA and SMX. This might be due to the lower log K_ow_ of SMX.

The separation factor defined by the Langmuir model was R_l_ = 1/(1 + K_l_C_0_), where C_0_ is the initial concentration of the solution, and the initial concentration of the mixed solution was 50–500 μmol·L^−1^, calculated as 0 < R_l_ < 1.Therefore, the adsorption process of the endocrine disruptors in the mixed solution by the hybrid microspheres was a preferential adsorption process. According to the results of the Freundlich model fitting, it is known that 0 < 1/n < 1, indicating that the adsorption process was relatively easy to perform.

The ΔG^0^, ΔH^0^, and ΔS^0^ in Langmuir thermodynamic fitting were calculated as shown in Table 5. It can be seen from Table 5 that the free energy of the hybrid microspheres in the adsorption process of endocrine disruptors was negative, and the value was about −21.5 kJ mol^−1^, indicating that the whole adsorption was spontaneous, and the process of physical adsorption was the main process. Moreover, as the temperature increased, the absolute value of the free energy slightly increased, indicating that the temperature had little effect on the entire adsorption process. In Table 5, ΔH^0^ was a negative value, indicating that the adsorption process was an exothermic reaction. ΔS^0^ was a positive value, indicating that the endocrine disruptor in the hybrid microsphere adsorption mixed solution was a process with an entropy increase. This indicates that the hybrid microsphere preferentially adsorbed BPA in the mixed solution.

### 3.8. Regeneration

**Figure 8:**
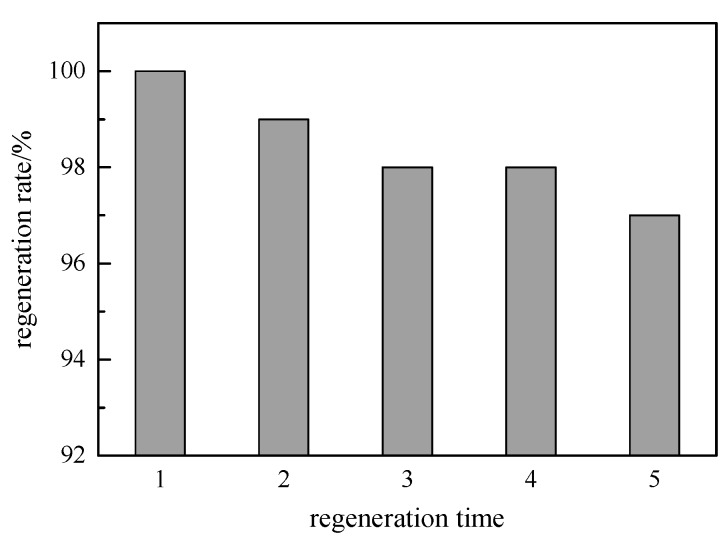
Regeneration effect of hybrid microspheres after adsorption.

The hybrid microspheres after adsorption were regenerated with a pure ethanol solution, and the results are shown in Figure 8. After five regenerations, the removal effect of the hybrid microspheres on the endocrine disruptors remained above 95%. This is because the endocrine disruptors were not strong at the adsorption site, and they were easily resolved. This result suggests that the hybrid microspheres could be repeatedly used, thereby reducing the cost.

## 4. Conclusions

(1) The effects of hybrid microspheres on the adsorption of BPA and SMX were comprehensively investigated. The increase in doping ratio was beneficial for increasing the adsorption amount of hybrid microspheres to BPA and SMX. The modified attapulgite played a major role in the adsorption process of endocrine disruptors, while PSE acted as a skeleton support embedding modified bump. The increase in pH was beneficial for increasing the adsorption amount of hybrid microspheres to BPA and SMX in the mixed solution. The pH mainly affected the SMX adsorption but had little effect on the BPA adsorption. Hybrid microspheres had a stronger adsorption effect on anionic endocrine disruptors than cationic ones. The hybrid microsphere had a better affinity for the hydrophobic endocrine disruptor. When the initial concentration of endocrine disruptors increased, the amount of adsorption of the endocrine disruptors by the hybrid microspheres increased. Hybrid microspheres preferentially adsorbed BPA, since some of the SMX adsorption sites were occupied by BPA molecules.

(2) Adsorption kinetics studies showed that the endocrine disruption of hybrid microspheres reached equilibrium within four days. The adsorption process was more in line with the quasi-second-order kinetic model, and the theoretically calculated equilibrium adsorption amount was close to that obtained by the experimental equilibrium. The rate constant K_2_ in the quasi-secondary kinetics decreased with the increase in initial concentration of endocrine disruptors due to the limited adsorption sites provided by a certain amount of hybrid microspheres. According to the results of the intraparticle diffusion model fitting, the adsorption process could be divided into two stages. Phase I was a macroporous diffusion process, which was completed in about 6 h, and the adsorption proceeded rapidly, whereas phase II was a diffusion process within the particle, and adsorption equilibrium was achieved around four days.

(3) The adsorption thermodynamic analysis showed that the adsorption process of BPA and SMX in the hybrid solution was more consistent with the extended Langmuir adsorption thermodynamic model. The process of hybrid microsphere adsorption of BPA + SMX was more consistent with the Langmuir adsorption thermodynamic model. Therefore, the adsorption process was uniform monolayer surface adsorption, and there was competitive adsorption. The maximum adsorption amount of the hybridized microspheres to the BPA was greater than the maximum adsorption amount to the SMX. It was a spontaneous, entropy-increasing, and exothermic reaction process based on physical adsorption, making it easier to carry out the preferential adsorption process. The adsorption process was less affected by temperature.

(4) After five regenerations, the removal effect of the hybrid microspheres on the endocrine disruptors in the mixed solution remained above 95%.

## Figures and Tables

**Table 1:** Hybrid microsphere diameter, specific surface area, porosity, and total micropore volume. PES—polyethersulfone.

Parameter	PA1	PA2	PA3	PA4	PA5
Modified attapulgite:PES	0:1	0.5:1	1:1	1.5:1	2:1
Diameter (mm)	2.41 ± 0.12	2.74 ± 0.14	2.71 ± 0.15	2.98 ± 0.13	2.95 ± 0.11
Specific surface area (m^2^∙g^−1^)	10.21 ± 0.13	22.17 ± 0.18	34.77 ± 0.21	46.32 ± 0.15	53.06 ± 0.19
Porosity (%)	80.21 ± 0.13	76.21 ± 0.15	75.49 ± 0.13	74.33 ± 0.14	73.21 ± 0.14
Total pore volume (cm^3^·g^−1^)	5.21 ± 0.13	2.81 ± 0.13	2.55 ± 0.14	2.13 ± 0.11	1.82 ± 0.12

**Table 2:** Fitting parameters of quasi-first-order and quasi-second-order kinetic models under different mixed solution concentrations.

C_0_ µmol·L^−1^	q_e,exp_ µmol·g^−1^	Quasi-First-Order Dynamics			Quasi-Secondary Dynamics		
		q_e,cal_ µmol·g^−1^	k_1_ h^−1^	*R_1_* ^2^	q_e,cal_ µmol·g^−1^	k_2_ g·(µmol∙h)^−1^	*R_2_* ^2^
50	8.91	3.92	−0.053	0.8881	9.02	0.046	0.9991
100	17.11	8.06	−0.064	0.8634	17.36	0.027	0.9998
150	25.98	13.06	−0.031	0.9617	26.32	0.0090	0.9980
200	32.80	21.76	−0.042	0.9157	33.67	0.0058	0.9965
250	39.87	26.46	−0.045	0.8932	40.98	0.0048	0.9971

**Table 3:** Fitting parameters of intraparticle diffusion model under different mixed solution concentrations.

C_0_ µmol·L^−1^	q_e,exp_ µmol·g^−1^	Phase Ⅰ		Phase Ⅱ	
		k_p1_ µmol·h^−0.5^·g^−1^	*R_p1_* ^2^	k_p2_ µmol·h^−0.5^·g^−1^	*R_p2_* ^2^
50	8.91	1.06	0.9953	0.28	0.9927
100	17.11	4.01	0.9928	0.35	0.9924
150	25.98	5.49	0.9988	1.05	0.9991
200	32.80	7.24	0.9985	1.56	0.9974
250	39.87	9.40	0.9990	1.93	0.9977

**Table 4:** Thermodynamic model fitting parameters of binary mixed solution. BPA—bisphenol A; SMX—sulfamethoxazole.

Category	T (K)	Langmuir Model			Freundlich Model			Extending the Langmuir Model		
		q_m_ µmol·g^−1^	K_L_ L·µmol^−1^	*R* ^2^	K_F_ µmol^1−n^·L^n^·g^−1^	*n*	*R* ^2^	q_m_ µmol·g^−1^	K_L_ L·µmol^−1^	*R* ^2^
BPA	298	102.04	0.021	0.9948	2.96	1.31	0.9872	112.98	0.019	0.9974
	313	97.09	0.020	0.9974	2.73	1.33	0.9898	108.18	0.018	0.9978
	328	90.91	0.018	0.9905	2.38	1.34	0.9886	106.46	0.015	0.9954
SMX	298	12.80	0.029	0.9976	1.36	2.42	0.9028	14.98	0.023	0.9991
	313	10.73	0.014	0.9924	0.55	1.95	0.9795	10.39	0.014	0.9962
	328	8.35	0.0077	0.9939	0.19	1.60	0.9821	6.83	0.0097	0.9972
BPA + SMX	298	112.36	0.0052	0.9917	1.36	1.40	0.9833	\		
	313	101.02	0.0043	0.9996	0.97	1.39	0.9898			
	328	99.01	0.0038	0.9959	0.78	1.35	0.9921			

**Table 5:** Thermodynamic parameters of binary mixed solution.

T (K)	ΔG^0^ (kJ∙mol^−1^)	ΔH^0^ (kJ∙mol^−1^)	ΔS^0^ (J∙mol^−1^∙K^−1^)
298	−21.24	−5.62	62.89
313	−21.78		
328	−22.23		

## Abbreviations

EDCs	endocrine-disrupting compounds
PCB	polychlorinated biphenyl
AP	alkylphenol
BPA	bisphenol A
APE	alkylphenol ethoxylate
PAE	phthalate;
PES	polyethersulfone
DMAC	*N*,*N*-dimethylacetamide solvent
SMX	sulfamethoxazole
CTMAB	cetyltrimethylammonium
AH-ATT	acid heat-modified attapulgite
OAT-ATT	organic acid heat-modified attapulgite
